# Exploring Older Adults’ Enhanced Activities of Daily Living

**DOI:** 10.1093/geroni/igaf122.3766

**Published:** 2025-12-31

**Authors:** Hye Soo Lee, Varitnan Hattakitjamroen, Hannah Morrow, Michael Bixter, Tracy Mitzner, Laura Payne, Wendy Rogers

**Affiliations:** University of Illinois-Urbana-Champaign, Champaign, Illinois, United States; Champaign, Illinois, United States; Montclair State University, Montclair, New Jersey, United States; Montclair State University, Montclair, New Jersey, United States; Person in Design, Atlanta, Georgia, United States; University of Illinois-Urbana-Champaign, Champaign, Illinois, United States; University of Illinois-Urbana-Champaign, Champaign, Illinois, United States

## Abstract

The concept of Enhanced Activities of Daily Living (EADL) recognizes that older adults engage in activities that go beyond the basic and instrumental ADLs. EADLs contribute to health, well-being, and social engagement, which are critical for successful aging. However, they are understudied using the ADL framework, including what older adults consider EADLs. We conducted an online survey wherein participants chose four EADLs from 31 activities (e.g., arts and crafts, working, hiking) developed based on the literature and prior interviews. Then, they rated their satisfaction and challenges for each activity (1 = strongly disagree to 5 = strongly agree). The final sample was 106 participants (age M = 70.6, SD = 6, range=60-92) who were 66% female, 86% with a bachelor’s or higher degree, and 87% White. Half had an impairment in vision, hearing, mobility, or cognition (53%). Most participants chose from the activities provided, with only 14 of 424 responses (3%) selecting others. We identified two new activities from the open-ended responses for others (family time, spiritual or religious). The most frequently chosen activities were exercise (n = 35), followed by reading (n = 28), volunteering (n = 28), and music (n = 27). Participants were generally satisfied (M = 4, SD = 0.5) and reported few challenges to engaging in EADLs (M = 1.8, SD = 0.5). In conclusion, despite the diversity in our sample’s functional capacity, our activity list was comprehensive, and participants showed high EADL satisfaction. These data provide insights about the range of activities that contribute to the quality of life for older adults and will guide the development of an EADL satisfaction measurement scale.

